# On the correlation between second order texture features and human observer detection performance in digital images

**DOI:** 10.1038/s41598-020-69816-z

**Published:** 2020-08-11

**Authors:** William H. Nisbett, Amar Kavuri, Mini Das

**Affiliations:** 1 Department of Physics, University of Houston, Houston, TX 77004 USA; 2 Department of Biomedical Engineering, University of Houston, Houston, TX 77004 USA

**Keywords:** Biomedical engineering, Translational research

## Abstract

Image texture, the relative spatial arrangement of intensity values in an image, encodes valuable information about the scene. As it stands, much of this potential information remains untapped. Understanding how to decipher textural details would afford another method of extracting knowledge of the physical world from images. In this work, we attempt to bridge the gap in research between quantitative texture analysis and the visual perception of textures. The impact of changes in image texture on human observer’s ability to perform signal detection and localization tasks in complex digital images is not understood. We examine this critical question by studying task-based human observer performance in detecting and localizing signals in tomographic breast images. We have also investigated how these changes impact the formation of second-order image texture. We used digital breast tomosynthesis (DBT) an FDA approved tomographic X-ray breast imaging method as the modality of choice to show our preliminary results. Our human observer studies involve localization ROC (LROC) studies for low contrast mass detection in DBT. Simulated images are used as they offer the benefit of known ground truth. Our results prove that changes in system geometry or processing leads to changes in image texture magnitudes. We show that the variations in several well-known texture features estimated in digital images correlate with human observer detection–localization performance for signals embedded in them. This insight can allow efficient and practical techniques to identify the best imaging system design and algorithms or filtering tools by examining the changes in these texture features. This concept linking texture feature estimates and task based image quality assessment can be extended to several other imaging modalities and applications as well. It can also offer feedback in system and algorithm designs with a goal to improve perceptual benefits. Broader impact can be in wide array of areas including imaging system design, image processing, data science, machine learning, computer vision, perceptual and vision science. Our results also point to the caution that must be exercised in using these texture features as image-based radiomic features or as predictive markers for risk assessment as they are sensitive to system or image processing changes.

## Introduction

Texture is a fundamental aspect of all images that describes the spatial arrangement and underlying structure of intensity values. Similar to color, texture encodes essential information about both an image’s subject, scenery, digital noise as well as the interaction between these. Deciphering the subtleties of texture would afford another layer of detail about images and provide a valuable perspective from which they could be characterized. In particular, the importance of image texture in the human visual perception and the ability of observers to perceive differences in textures have been studied extensively. Much of the fundamental work on image texture analysis itself was carried out in the field of visual perception by the likes of Julesz, who conducted research on the role of texture in perception from a statistical perspective^1^. His findings in particular called attention to the importance of image texture and catalyzed a great amount of research on the subject outside of vision science.

In an effort to further understand the information that image texture holds, others outside of perception sought to capture the essence of different textures and quantify them into so-called features. As a result, image texture features grew into an extensive class of metrics capable of quantifying complex image attributes and textural patterns. The works of Haralick^2,3^ in particular proved influential in quantitative texture analysis and his Grey Level Co-occurrence Matrix (GLCM) method of texture analysis is still widely used today. His as well as other’s seminal works in quantitative texture analysis helped spark a field of research that has seen substantial growth in recent years thanks to improved computational power and a renaissance in machine learning^4^.

Though much of the early research in quantitative texture analysis, including Haralicks’, was conducted in remote sensing^5–9^, texture analysis techniques have since disseminated into several fields including materials science, geoscience, and medical imaging. In medical imaging in particular, texture features have found a variety of applications including diagnostic imaging, risk assessment, and radiomics^10–14^. Second-order texture statistics, such as Haralick’s GLCM and Galloway’s grey level Run Length Matrix (RLM)^7^, have been used extensively to these effects.

A significant body of texture analysis related work in medical imaging has been in the area of Digital Breast Tomosynthesis (DBT), computed tomography, and Digital Mammography (DM). DBT is a limited angle tomographic X-ray imaging technique that allows volumetric imaging of breast. Parenchymal texture analysis in breast imaging has been an active field of research. Caldwell et al.^15^ and Tahoces et al.^16^ both suggested that parenchymal texture features could be used to assess breast cancer risk, similar to how breast density has been used. Huo et al.^17^, Li et al.^18,19^ all built upon these results and worked to identify texture features that are useful discriminators of low- and high-risk patients. Several others have since developed fully automated procedures for breast cancer risk assessment based off of parenchymal texture features in both DBT and Digital Mammography DM^20–22^.

Using texture features as predictors of risk relies on the features being able to accurately quantify structural information about the subject. However, research indicates that texture significantly varies not only between clinical systems and modalities, but also across the space of acquisition and reconstruction parameters^23–26^. In addition, the noise from the randomness in photon emission and detection processes also becomes a dominating factor. This makes disentangling structural and system-related contributions to texture features challenging, but also necessary for true quantitation. This is especially relevant for tomographic modalities, such as tomosynthesis or computed tomography (CT), where the anatomical information revealed in images depends heavily on acquisition and reconstruction settings. Therefore, an improved understanding of how system design impacts image texture is prerequisite to the development of robust quantitative models involving texture.

In addition to encoding detailed information about anatomical structures, texture features also impact our visual perception of structures and inhomogeneities in images. However, it is not well understood how changes in these texture features may correlate with human observer perceptual abilities and signal detection tasks. In medical imaging particularly, clinical tasks such as the detection of cancers are fundamentally related to the perception of abnormalities in a complex, textured background. As such, radiological performance ought to be closely linked to the textural properties of the image background in which the signal is embedded^27^. The benefits of understanding how these properties affect detection performance and recall rates are twofold: First, it will provide further insight into the human visual system and its response to texture. Second, it can potentially allow efficient mechanisms for the optimization of system geometries and reconstruction procedures for improved observer performance. In this way, texture features could form a new class of image quality metrics with clinical significance for detection–localization tasks in medical images^27^. Canonical metrics such as mean-squared error and signal-to-noise ratio struggle in this regard since they fail to accurately reflect perceived image quality, most importantly the task-based assessment of image quality^28–30^.

In this paper, we examine the correlation of several well known texture features computed on simulated DBT images with the ability of human observers to detect low contrast signals in them. Simulated images offer the benefit of known ground truth unlike in the case of clinical images. The goals of our study are twofold: First, we will investigate how texture features vary with respect to changes in DBT acquisition and filtering parameters. Second, we will examine how these same changes in image texture could potentially impact an observers’ abilities to locate a signal in DBT images. A correlation between the two can offer insight into the feasibility of using texture parameters for simple task-based assessment methods with potential for direct clinical applications. Here the assumption is that the observer is trained to recognize the signal type (cancer features etc.) if the signal is visible to them—true for trained radiologists or for the case when the observer is aware of the signal type, average size and shape.

Based on the results of several studies, Julesz concluded that first- and second-order texture statistics appear to be immensely important in guiding the attention of observers to certain textured regions^1,31–33^. It was also shown that humans had difficulty in classifying images with similar second order textures. With this in mind, we have elected to analyze second-order texture features in this study, though other classes of features may also yield impactful findings. We vary the quantum and anatomical noise properties in the study sets by changing the sampling rates and filtering in tomographic imaging. These new results would enhance and compliment our ongoing work to develop visual search observer models for radiological imaging^34–36^.

## Methods and results

In order to examine the dependence of image texture features on human observer detection performance in digital images, we used DBT images generated from a realistic computer simulation platform (described in section Image Generation below). The platform allows the capability to generate image sets with different acquisition or processing strategies. The 2D image slabs extracted from the generated volumetric images were then presented to human observers to assess their detection-localization performance (see section Human Observer LROC Studies below). Further, these exact same image slabs were used in estimating texture features described in section entitled Texture Analysis below. In subsequent sections, we present the details of the observed correlation of texture features with human observer detection–localization performance.

### Image generation

We used an analytical approach to model the entire imaging chain which includes models for breast tissue structures (to mimic breast structure complexity in a patient population). A similar approach can be found in several of our past publications^37–39^. Monte Carlo simulation methods can also be used to generate similar realistic virtual images when digital breast models are used. However, these can be time consuming to generate several study sets. We will describe some key elements including breast models and the virtual X-ray image generation process.

**Figure 1:**
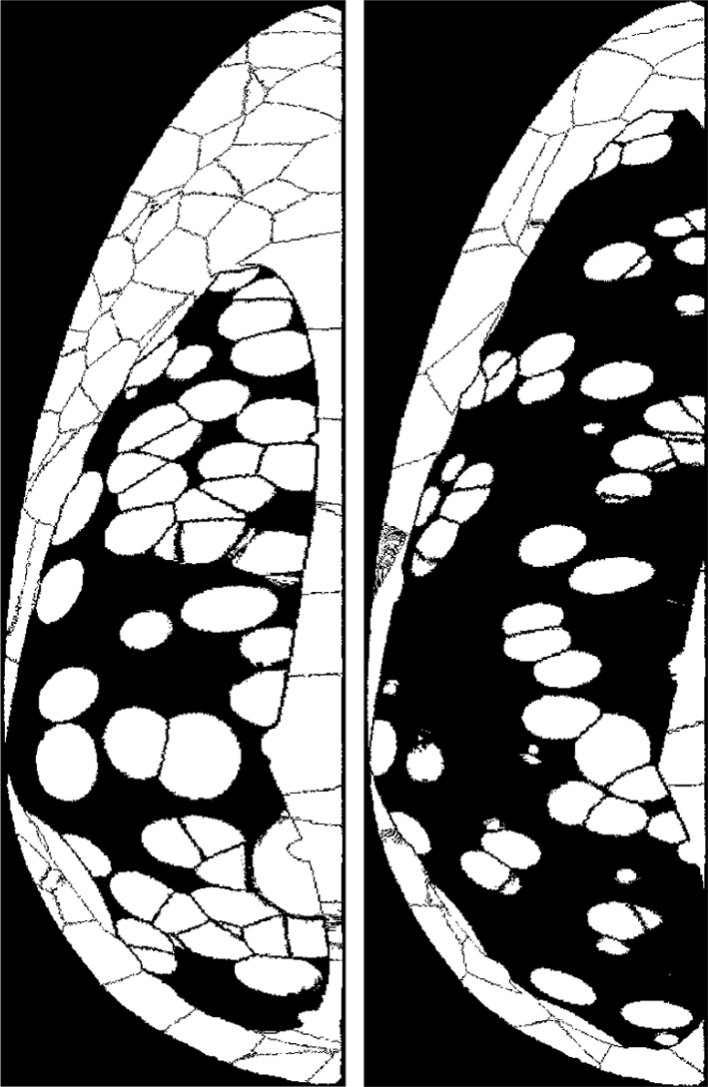
Sample slices of 25% dense (left) and 50% dense (right) phantoms composed of adipose, fibroglandular tissue, skin, and Cooper’s ligaments.

Breast phantom models have been generated by several groups^40–43^ to allow virtual breast imaging using multiple modalities. In our study, we imaged virtual, anthropomorphic compressed breast phantoms generated by Bakic et al.^44^ under various acquisition geometries. Bakic phantoms are computer generated voxelized 3D phantoms. When these phantoms are used in realistic X-ray imaging simulations, the resulting images adequately mimic the complexity of clinical mammograms and tomographic breast images. The digital phantoms used in this study are of 5 cm uniform thickness to mimic the typical compressed breast in mammography or DBT. The phantom model is composed of adipose and fibroglandular compartments separated by Cooper’s ligaments. The voxels corresponding to these compartments are assigned appropriate tissue attenuation properties. This phantom design allows variations in glandularity, shape and size of compartments, resulting in sufficient structural variability among phantoms—similar to the expected variability in a patient population. It also allows change in phantom density by varying dense tissue compartments in the fibroglandular region. Sample Bakic phantom slices of two different densities are shown in Fig. 1.

The simulation platform used in this study to generate DBT images is detailed in reference^37^ and our other publications^35,45–47^. A serial cascade analytical model is used to emulate X-ray projections through the phantoms, followed by a tomographic reconstruction for any chosen DBT system geometry. Siddon’s ray-tracing algorithm is implemented to model X-ray transmission through the voxelized (with 3D voxel size of $0.2\,{\text {mm}}\times 0.2\,{\text {mm}}\times 0.2\,{\text {mm}}$) phantom^48^. This includes source spectrum, X-ray projection through digitized 3D object phantom, detector gain, noise and system blur. The models account for blurring effects due to both the focal-spot and the scintillator component of the detector. The detector in this study was modeled as a CsI (0.1 mm thick) based a:Si flat panel detector. Quantum detection efficiency, scintillator gain, and optical collection efficiency are also considered in the model. Noise propagation through the detector was modeled as a scaled Poisson process at each energy interval along with additive electronic noise. A Feldkamp filtered back projection (FBP) algorithm was used for image reconstruction from these generated projections. This was followed by a 3D Butterworth filter (0.25 cycles/pixel cut-off frequency). For each reconstructed volume, a slabbing procedure was implemented to produce 1-mm thick image slabs as in clinical DBT imaging. Each slab has a dimension of $760\times 240$ with in-plane resolution of $0.27\,{\text {mm}}\times 0.27\,{\text {mm}}$.

**Figure 2:**
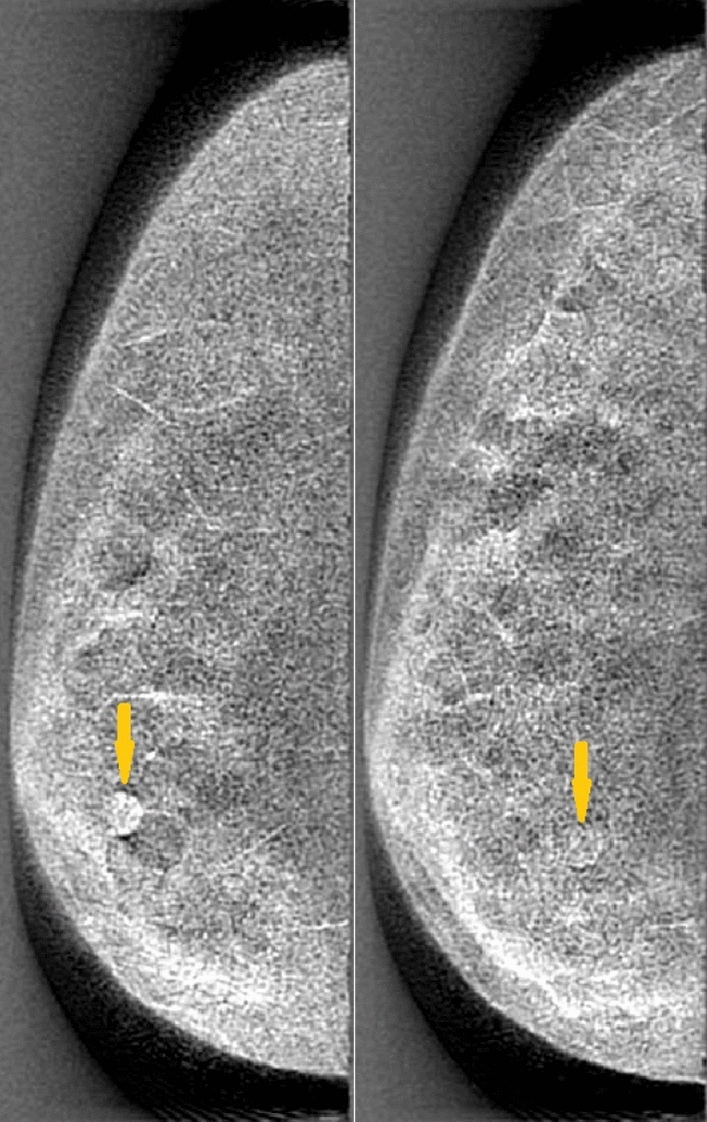
Sample DBT images of 25% (left) and 50% (right) dense digital phantoms, both taken across a span of $60^{\circ }$ and reconstructed using 21 projections. Lesions are present in the bottom-left and bottom-middle of left and right phantoms, respectively.

The presence of low-contrast cancer mass in the breast volumes was emulated by inserting spherical signal in the breast model prior to the projection step described above. These digitized homogeneous spherical lesion models (of 8 mm diameter) were randomly inserted at a specified depth in glandular volume after the corresponding background tissue is removed. The energy dependent attenuation properties of these lesions were chosen to model those of infiltrating ductal carcinoma based upon prior empirical measurements^49^. Thus, following image reconstruction, the visibility of these lesions in the generated DBT images have a realistic level of perceptual difficulty as in a clinical DBT image. In order to mimic the varying levels of difficulty encountered in breast radiology, breasts of different densities were used in the study. Half of the phantoms were designed with a Volumetric Glandular Fraction (VGF) of 25% and the other half with a VGF of 50%. While VGF of 50% is higher than the average breast densities found in patients, we chose this distribution to add sufficient range of difficulty in the detection task. Figure 2 provides a comparison of DBT images using two phantoms of different densities with inserted lesions that were imaged and reconstructed under the same conditions.

In order to examine signal detectability in human observer studies, image slabs with and without lesion were generated. To generate lesion-present cases, digital lesion models were inserted in the fibroglandular compartment of the breast phantom and these had voxel size matching that of the breast phantom, $0.2\,{\text {mm}}\times 0.2\,{\text {mm}}\times 0.2\,{\text {mm}}$. While each breast phantom imaging resulted in a single 3D image volume, our detection studies involved presenting only the 2D slabs/slices to the human observers. As a result, we were able to utilize a scheme (described below) that allowed significant increase in the number of unique and independent images obtainable from just six 3D breast phantoms.

The visibility of inserted lesion in an image is impeded by the density and structural variations of the surrounding tissue. In addition, the glandular structure varies throughout the breast volume. As a result, inserting the lesions at different phantom depths for each independent projection run allowed us to extract multiple unique image slices for the perception study even from a single phantom. Accordingly, the image slabs corresponding to eight lesion-present locations could be treated as eight independent cases (even if obtained from a single 3D breast phantom) in a given study set where perceptual questions are examined. Eight lesion-present variants of each phantom were created by randomly inserting a voxelized lesion at different depths in the breast volume. Similarly, a ninth imaging run was conducted using the same phantom with no inserted lesions. Thus eight image slabs with lesion-present were extracted from the first eight runs and an additional eight image slabs were extracted with no lesions from the ninth run (totaling 16 unique images from a single breast phantom). This allowed sufficient variability perception study design when using these slabs extracted from the same 3D breast phantom. Thus for a chosen image acquisition and processing method, a total of 96 unique 2D images were generated from the available 6 breast phantom volumes ($16\,{\text {slabs}}\times 6\,{\text {phantoms}}$).

**Figure 3:**
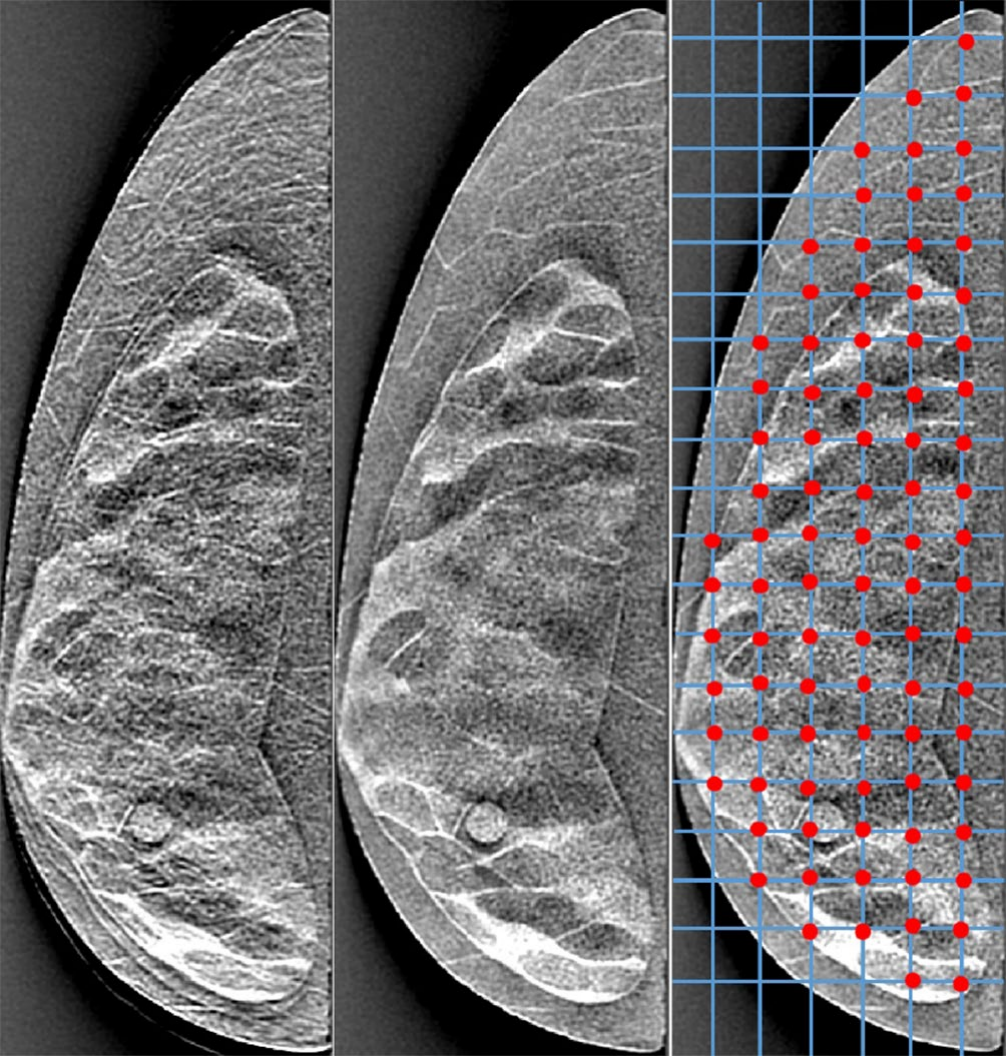
An example of three simulated DBT images used in our study. Each image is of the same 25% dense phantom taken over a $60^{\circ }$ span with varying numbers of projections (from left to right: 3, 19, and 51). The target lesion can be found in the bottom-middle portion of each image. Outlines of the ROIs used for texture analysis can be found in the rightmost image. The red dots helps identify the ROIs within the breast region used for texture analysis.

To evaluate changes in signal detectability with changing DBT imaging geometry, we generated images by changing the number of projections for a chosen arc angle. Each study set consisted of a number of equally spaced projections, *P*, acquired over a $60^{\circ }$ angular span of the phantom. A total of 12 cases were considered, where the number of projections $P \in \{3, 7, 11, 15, 19, 21, 25, 31, 35, 41, 45, 51\}$. For each of these simulations, a total dose of 1.5 mGy was evenly distributed across the *P* projections. Thus the number of unique slices used in the study becomes 1152 (96 slabs $\times$ 12). We present a comparison of DBT images reconstructed using different numbers of projections in Fig. 3. We show single slab which had the lesion from each DBT volume. Simple visual inspection indicates that increasing the number of projections used in the reconstruction helps to resolve reconstruction artifacts and improve perceived visual quality up to a certain point. A corresponding set of image slices with Wiener filtering (described below) applied in projections were generated to examine the effect of filtering in signal detection, yielding a total of 2304 image slices ($1152 \times 2$) used in the complete study. Note that the grids in the right-most image in Fig. 3 is relevant only in regards to the lattice selection in texture analysis methods referred in the sections below.

### Wiener filter

**Figure 4:**
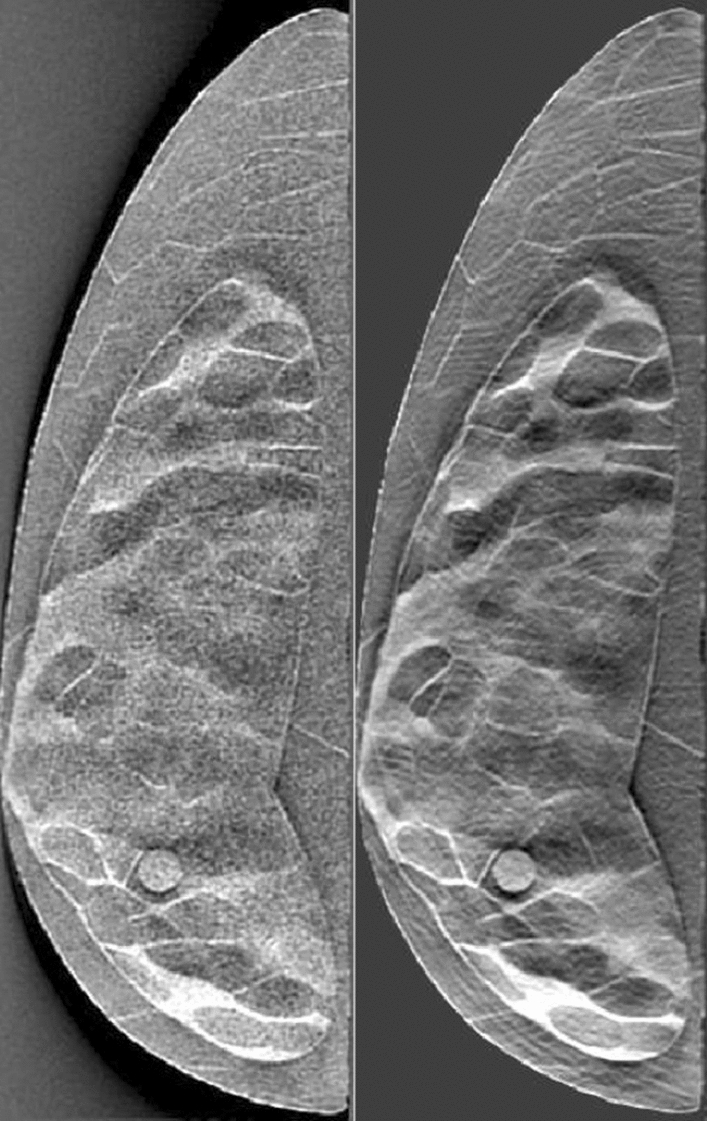
An unfiltered DBT image (left) of a 25% dense phantom and the same image after application of the Wiener filter (right). The unfiltered image was acquired across a $60^{\circ }$ span with 15 projections.

In DBT, as the total dose is distributed across multiple projections acquired over a limited arc span, the amount of quantum noise increases. In the prior work by Vieira et al.^50^, an adaptive Wiener filter was shown to reduce the noise in DBT images without deteriorating actual structures and improve micro-calcification detection performance. The Wiener filter was designed to reduce additive Gaussian noise by minimizing the mean square error. The filter used in this study is a space-variant version applied on the DBT projections. The details of these can be also seen in our recent work^51,52^. As described above, we applied the filter to the noisy projections to generate a set of 1152 filtered images. This filtered set of images were used for a separate human observer study and texture analysis (both described below) to examine the effect of image filtration. In Fig. 4, we present a comparison of reconstructed images before and after applying Wiener filter.

### Human observer LROC study

Once an ensemble of breast images are generated (as described in the above sections) using multiple acquisition and processing techniques, one can now test the detection benefits of these techniques using human observer detection studies. In this section, we describe the methods and results of our human observer studies, which is used as a gold standard to assess benefits of acquisition or processing protocols. The protocols tested include detection of performance changes with the total number of projections for a fixed 60° arc in DBT. A dose of 1.5 mGy was equally distributed among the projections for each case. We also examined these same acquisition protocols when an adaptive Weiner filtering was applied to the projections prior to tomographic reconstruction.

Low contrast lesion detection and identification, is an important task in screening and diagnostic breast imaging. Classically, Receiver Operating Characteristic (ROC) analysis has been used to perform task-based assessment of observer performance for such radiological tasks. The results of this analysis can then be used as a proxy for image quality. In this study, we use a modified ROC analysis known as Localization ROC (LROC) in order to assess human observer performance in both detection and localization of low-contrast mass detection in a structurally complex and noisy anatomical background. Briefly, observers are presented with the slabbed DBT images and are tasked with classifying the images as either “lesion-present” or “lesion-absent” and assigning confidence ratings of either “low” or “high” to their choices. In addition, to be counted as accurate diagnosis, if an observer classifies an image as “lesion-present”, they must also localize the lesion to within a 15 pixels distance of its center. For any decision, the observer has the option to pick from a range of confidence levels on a four point scale (with 1 being low and 4 being high confidence) and the option to mark the most probable lesion location in the image. These choices are recorded for the experimenter to later use for analysis. Additional details of our human observer studies can be found in our prior publications^38,39^.

Three non-radiologist observers participated in two separate LROC studies, one for images with a Wiener filter^50^ applied and one for images without. In each study, the observers were presented with images of both $25\%$ and $50\%$ dense phantoms obtained across an angular span of $60^{\circ }$ and reconstructed using some number of projections $P \in \{3, 7, 11, 25, 45\}$. In each session, observers were first presented with 18 training images followed by 54 test images some of which had randomly placed lesions (the locations of which are only known to the experimenter and not the observer). Each observer completed two sessions for each *P*. Observer performance was quantified as the Area Under the LROC Curve (AUC), which was estimated using the Wilcoxon rank-sum test statistic.

### LROC study results

**Figure 5:**
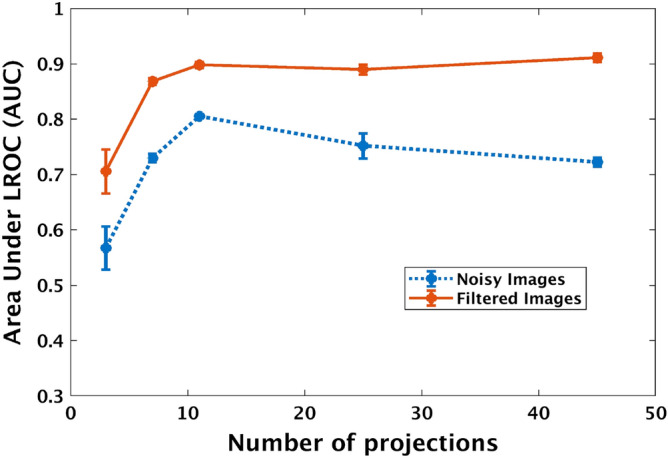
Area under the LROC curve (AUC) for human observer detection of a low contrast mass in DBT images. Shown here with changing number of projections and post processing for a 60° arc DBT. Each error bar represents the standard error in the mean AUC at that number of projections based on the three observers. Correlation of these results with texture properties estimated from these same images are presented in the sections below.

We conducted two LROC studies for the subset of $60^{\circ }$ as detailed in the above section and computed the AUC for each trial. The mean AUC of the three observer performances were plotted as a function of the number of projections in Fig. 5. For low projection numbers, the image degradation is primarily due to artifacts. This is resolved with increasing projection numbers (for the same total dose for each study set). However as the number of projections increases, quantum noise increases due to fewer photon counts per projection. Addressing first the observer performance on noisy images, we find that at low projections number, observer performance benefits from the addition of more projections, up to a certain limit. After this limit, observer performance declines as further projections are added. One also observes that the average AUC improves for all study sets (chosen number of total projections for the 60° arc) when adaptive Weiner filtering is applied to the projections prior to tomographic reconstruction. This indicates that observers were able to more successfully and confidently localize the lesions in these filtered images. Furthermore, we notice that observer performance for this image set still experiences an increase at low projections number; however, it no longer decreases at higher projections as it did in the unfiltered image set. Thus one can imply that the adaptive Wiener filtering was able to successfully reduce the quantum noise without degrading the relevant structural details. The image slabs used in human observer studies were also used in texture analysis (described below).

### Texture analysis

We remind the reader that the main goal of this paper is to identify image texture features that inhibit or enable signal detection in digital tomographic images. This can be also understood by examining the correlation between the changing magnitude of some average texture features within the images and changes in human observer detection performance as we vary system or filtering operations. In the above sections, we described our image generation methods, human observer LROC studies and the results for changing DBT projection numbers and image filtration. Next, we describe our texture analysis methods and the results for changing texture values with changes in DBT parameters described above. Finally, we will examine the correlation between these texture parameters and observer performance. Texture feature analysis was performed using codes developed in-house using MATLAB 2016b (https://www.mathworks.com) and Python v3.7 (https://www.python.org).

#### Quantization and ROI selection

The DBT image slabs used in human observer studies were also used for texture analysis. Briefly, we first quantized each slabbed DBT image into 256 grey levels for computational efficiency. Next, the entire breast region was subdivided into lattice Regions of Interest (ROIs). We then computed 13 texture features in each ROI based on three well documented second-order texture matrices. Finally, we averaged each texture results of the all the ROIs across the images for a chosen study case. As an example, for a given ’number of projections’ *P* in the DBT image data.

For the results shown here, each ROI measured 35-by-35 pixels ($9.45\,{\text {mm}}\times 9.45\,{\text {mm}}$). We chose the ROI to be slightly larger than the signal size of interest—which was spherical with 8 mm diameter, contained roughly within 30 by 30 pixels in an image slab. We examined three texture metrics used widely in literature namely: Grey level co-occurrence matrix (GLCM), Neighborhood grey tone difference matrix (NGTDM), and Grey level run length matrix (RLM). These are detailed below. Out of several possible texture feature calculation, we chose four GLCM features, four NGTDM features and five RLM features as these are found to be used extensively in vision science research for image discrimination tasks^1^.

#### Grey level co-occurrence matrix

We computed normalized GLCMs according to Haralick’s original method^2^. An image’s GLCM describes the probability of two grey levels co-occurring at some fixed distance and direction from one another for the entire image. Based on the literature, we chose the separation between the two pixels, *d*, to be 1 and the directions to be $0^{\circ }, 45^{\circ }, 90^{\circ }, 135^{\circ }$. This results in four unique GLCMs for each ROI.

Four texture features were calculated according to Haralick’s original definitions as listed in Table 1: Energy, Entropy, Homogeneity (referred to by Haralick as “inverse difference”), and Correlation. Many of the GLCM features have intuitive and familiar definitions. For instance, Energy is the second moment of the GLCM. The texture feature Entropy is defined by applying the standard definition of entropy to the co-occurrence distribution. It is therefore related to the expected rate at which information is gained through sampling the distribution; lower values of Entropy correspond to a higher expected rate of acquiring information.

**Table 1:** Definitions of four GLCM statistics that were utilized in this study.

Type	Feature	Definition
GLCM	Correlation	$\sum _{i,j}\frac{(ij)G(i,j) - \mu _x \mu _y}{\sigma _x\sigma _y}$
	Homogeneity	$\sum _{i,j}\frac{G(i,j)}{1 + |i-j|}$
	Energy	$\sum _{i,j}G(i,j)^2$
	Entropy	$- \sum _{i,j}G(i,j)log_2(G(i,j))$

Where *G* is the GLCM matrix with indices *i* and *j* varying from 1 to maximum grey level $N_{grey}$, $\mu _x=\sum _{i,j}iG(i,j), \mu _y=\sum _{i,j}jG(i,j), {\sigma _x}^2=\sum _{i,j}(i-\mu _x)^2G(i,j),$ and ${\sigma _y}^2=\sum _{i,j}(j-\mu _y)^2G(i,j)$

#### Neighborhood grey tone difference matrix

We also consider the Neighborhood Grey Tone Difference Matrix (NGTDM), as many others have in the literature on second-order texture analysis. The NGTDM is a vector describing how each grey level differs from its surrounding neighbors across an entire image^9^. The difference between each pixel of grey level *i* and the average of its surrounding neighborhood is computed and then summed across all pixels with the same grey level. The NGTDM takes a parameter, *n*, defining the size of the neighborhood. We have used $n = 1$ (i.e. we consider only immediately adjacent pixels) as Amadasun and King^9^ did.

From this vector, an additional four texture features namely Contrast, Coarseness, Busyness, and Complexity were computed according to their original definitions as shown in Table 2. Each of these features were designed by Amadasun and King from the perspective of visual perception and their mathematical definitions are meant to reflect this. Complexity, for instance, is designed to indicate the amount of visual information in an image. A texture with large number of rapid spatial variations such as edges and lines is more likely considered as complex compared to texture with local uniformity. This is achieved through mathematically considering the size and quantity of texture patches and basic patterns in an image. A full description of each of these features and the intuitions behind their definitions can be found in the original work.

**Table 2:** Definitions of four NGTDM features that were utilized in this study.

Type	Feature	Definition
NGTDM	Contrast	$\left[\frac{1}{N_g(N_g - 1)}\sum _{i,j}P_iP_j(i-j)^2\right]\left[\frac{1}{n^2}\sum _iD(i)\right]$
	Coarseness	$\left[\sum _iP_iD(i)\right]^{-1}$
	Busyness	$\left[\sum _i P_iD(i)\right]\diagup \left[\sum _{i,j}|iP_i - jP_j|\right]$
	Complexity	$\sum _{i,j}\{|i-j|\diagup (n^2P_i +\ n^2P_j)\}\{P_iD(i) + P_jD(j)\}$

Where *D* is the NGTDM vector, $p_i$ or $p_j$ represent the probability of occurrence of grey values *i* or *j* in an ROI, and $n^2$ is the number of center pixels used to estimate NGTDM.

#### Grey level run length matrix

The final matrix that we have computed from our images is Galloway’s RLM^7^. The RLM describes how frequently runs of each grey level occur for different lengths and image directions. This captures information about how fine or coarse each grey level’s textures are. More frequent, shorter runs may indicate finer textures while the opposite is true for coarser textures. Like the GLCM, the RLM requires a direction in which to compare pixels. Again we have used the directions $0^{\circ }, 45^{\circ }, 90^{\circ }, 135^{\circ }$ and averaged the RLM features calculated from the four directions matrices.

From each matrix, we computed the five features: Short Runs Emphasis (SRE), Long Runs Emphasis (LRE), Grey Level Nonuniformity (GLN), Run Length Nonuniformity (RLN), and Run Percentage (RP). Precise definitions and interpretations for each feature can be found in Galloway’s original work and also listed briefly in Table 3. One example is GLN, which takes on small values when runs are spread evenly across many grey levels; High values occur when an image’s runs are concentrated in a relatively small number of grey levels.

**Table 3:** Definitions of five RLM statistics that were utilized in this study.

Type	Feature	Definition
RLM	Short runs emphasis	$\frac{1}{N}\sum _{i,j}\frac{R(i,j)}{j^2}$
	Long runs emphasis	$\frac{1}{N}\sum _{i,j}j^2R(i,j)$
	Grey-level nonuniformity	$\frac{1}{N}\sum _i\left(\sum _jR(i,j)\right)^2$
	Run length nonuniformity	$\frac{1}{N}\sum _j\left(\sum _iR(i,j)\right)^2$
	Run percentage	$N\diagup P$

Where *R* is the run length matrix, *i* representing grey levels from 1 to maximum grey level $N_{grey}$, and *j* representing the run length from 1 to maximum run length $N_{run}$, *N* is total number of runs where N = $\sum _{i,j}R(i,j)$, *P* is the number of pixels in ROI.

## Results of texture analysis

The above described texture analysis was performed on all regions of interest for both the filtered and unfiltered DBT image sets, each of which were simulated under various conditions as described in the section titled Image Generation. We examined the changes in these texture features as a function of changing system parameters or the effect of image filtration. Subsequently, we evaluated the correlation of these texture feature changes with changes in human observer performance for the image sets under investigation. Human observer performance remains the the gold standard for assessing image quality in a task-relevant manner.

### Effect of sampling frequency and filtration on DBT image texture

**Figure 6:**
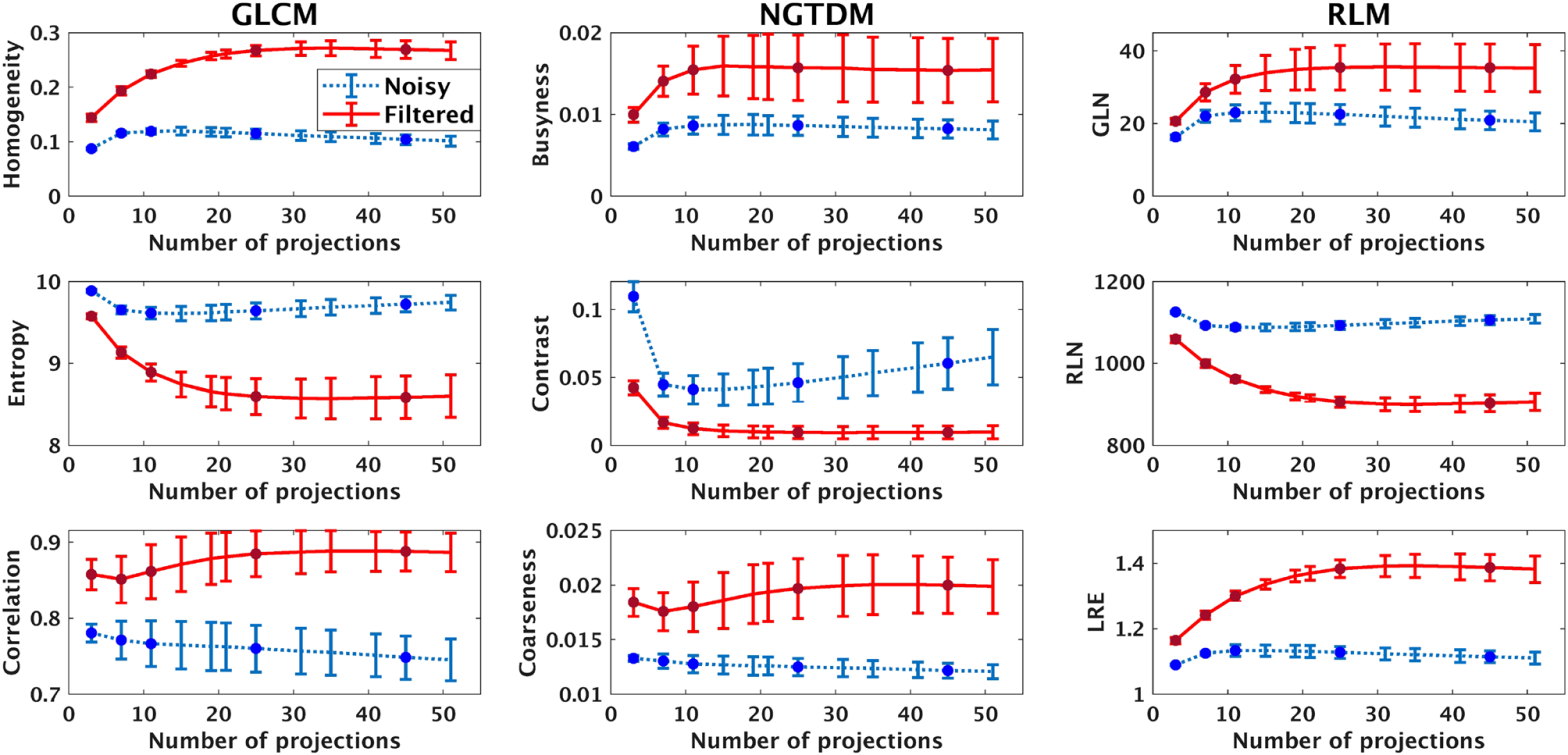
Mean values of several image texture features plotted as functions of number of projections for both noisy and Wiener filtered images. Each error bar represents the standard deviation in each direction of the mean texture value at that number of projections. The image set consisted of 25% and 50% dense phantoms imaged across a span of $60^{\circ }$.

In a tomographic breast imaging system, increasing the number of projections acquired over a given angular span amounts to increasing the angular sampling frequency of the phantom. Examining how this impacts texture features in our images affords valuable information about the behavior of the DBT system. We present the results of this question for nine texture features in Fig. 6. For brevity’s sake, we have only included enough plots to demonstrate the full spectrum of trends in the data. GLCM based parameters are shown in the left column, NGTDM parameters are shown in the middle column, and RLM parameters are presented in the right column. The first and second rows consist of features that show a very high correlation with trends in human observer performance with change in the number of DBT projections. The last row consist of features that show good or poor correlation. The texture response to change of number of projections is discussed first for noisy images.

Our results indicate that some of the texture feature curves show a trend similar to that of the trend exhibited by the human observer AUC values as the number of projections are changed (see Fig. 5). They can be generally characterized as having three key attributes: an initial nonlinear change (increase or decrease), a stationary point, and a subsequent linear retrogression. The initial change in image texture at lower projections number is likely driven by the improvement in reconstruction artifacts as the projection number increases. However, this increase is limited by the amount of new information contributed by the addition of projections, creating the stationary point. The physical mechanism responsible for the linear portion will be examined in the next section.

Two dominant trends exist in the texture features that we have tested, which we will refer to as “concave-up” and “concave-down”. Concave-down texture features are those whose second derivatives with respect to number of projections are generally negative (See features in the first row of Fig. 6). Likewise, concave-up features are those whose second derivatives are positive (See features in the second row of Fig. 6). Furthermore, amongst the features in each group, there appears to be a spectrum of different second-derivative magnitudes: some features reach stationary points quickly and then experience steep increases/decreases. Others do not reach their stationary points as soon and diverge more slowly. However, in all cases, it seems that after reaching their critical points, the texture features begin to retrogress in a linear fashion. Given our constant dose constraint, it is possible that the changes in image texture at higher projections number is related to the photon statistics of the reconstructed images. We will test this hypothesis in the next section using an adaptive Wiener filter.

### Texture changes with wiener filtering in DBT

In order to further strengthen the above results indicating strong correlation of several texture features with changes in DBT angular sampling, we examined correlations in texture features in these images with and without Wiener filtering. DBT images suffer an increase in quantum noise due to reduction in photon counts resulting from increasing sampling rate. Denoising due to Weiner filtering was effective in noise reduction while improving signal detection. The visual benefits of this was evident in Fig. 4, where we showed examples of image slabs with and without Weiner filtration in DBT. These benefits were also quantified in terms of improvements in the AUC for human observer studies (in the section above entitled “LROC Study Results”). Corresponding texture results for these denoised images are presented in Fig. 6. The texture features were computed using the Weiner filtered images as in the case of unfiltered images.

Based on these results, it appears that the Wiener filter does not impact the overall concavity of texture feature plots (i.e. concave-down features remain concave down and vice versa). However, many texture features’ trends did experience significant changes because of the filtering. Rather than reaching stationary points and then retrogressing, all features now remain relatively constant at higher number of projections and no longer show signs of retrogression. Furthermore, all features experienced noticeable changes in magnitude at each projections number because of the filter’s application. In the case of concave-down features, filtered-image texture feature magnitudes are larger than that of the unfiltered images. The opposite is true for concave-up features. Overall trends in these texture feature changes look similar to what is observed in the AUC changes with and without filtering (Fig. 5 above). In the section below, we estimate the correlation coefficients to quantify these similarities in trends.

### Correlation of image texture features with human observer performance

Comparing trends in human observer AUC changes with image texture features, we find most texture features appear to show a trend that correlates with human observer performance variations. It is of further significance to examine which parameters have stronger and more robust correlation with human observer detection performance. In order to quantify this correspondence, we computed Pearson correlation coefficients between each texture feature and our human observer data, which we present in Table 4. While we had images corresponding to 12 values *P* (total number of projections), human observer studies were conducted only using 5 sets (shown by the five points in each curve in Fig. 5. We estimated texture features for all 12 sets of data (as seen in 6). In these plots the solid dots corresponds to points for which human observer data is also available. The correlation between texture and human observer studies were conducted only using these five sets where both information (texture analysis and human observer data) was available. The correlation coefficients were estimated between the mean AUC values and mean texture values corresponding to these five sets with the number of projections $P \in \{3, 7, 11, 25, 45\}$. Comparing once more this AUC data with the texture features of these filtered images, we find that again a strong correlation exists between the two metrics. Several texture features maintain the high correlation as observed with human observer performance with unfiltered images.

Next, to further examine the robustness of texture features correlation with human observer detection–localization performance, we examined correlation coefficients using the complete set of data which also accounts for the change in relative performance from noisy to Wiener filtered images across change in the number of projects. Pearson correlation coefficients between the texture features and the AUC values were obtained when considering both the noisy and filtered images with changing DBT sampling. These results are reported in the ‘Noisy and filtered’ column of the Table 4. The texture features that showed higher correlation ($|r|>0.85$) with human observer performance under changes in system geometry and filtering operations are highlighted in the Table 4. As we see from these results, a subset of the features we presented retains high correlation with changing image artifacts and noise (due to projection sampling or filtering).

**Table 4:** Pearson correlation coefficients between texture features and observer performance. The features showing high correlation (and for all three columns) with human observer detection–localization performance are highlighted.

Texture feature	Noisy	Filtered	Noisy and filtered
*Pearson correlation coefficient (r)*			
GLCM			
**Entropy**	– 0.9727	– 0.9006	– 0.8813
**Homogeneity**	0.9529	0.8711	0.8603
Energy	0.9891	0.8434	0.8427
Correlation	– 0.5491	0.4853	0.6534
NGTDM			
**Contrast**	– 0.9660	– 0.9929	– 0.9597
**Complexity**	– 0.9662	– 0.9914	– 0.9588
**Busyness**	0.9756	0.9857	0.9081
Coarseness	– 0.4690	0.4719	0.653
RLM			
**Grey level nonuniformity**	0.9787	0.9431	0.9347
**Run length nonuniformity**	– 0.9547	– 0.8651	– 0.8573
**Run percentage**	– 0.9581	– 0.8562	– 0.8508
**Short run emphasis**	– 0.9527	– 0.8551	– 0.8500
Long run emphasis	0.9777	0.8457	0.8453

## Summary and discussions

We examined the relevance of changes in texture features in digital breast images with respect to the ability of human observers to detect and localize low contrast mass signals in these images. Using realistic simulated breast images, we examined the correlation coefficients between human observer detection–localization performance and texture features in these images. Visual inspection of the area under the LROC curves and the plotted texture features immediately reveals a great similarity in trends with changing imaging geometry and Wiener filtering. We chose three texture metrics GLCM, NGTDM and RLM, which are widely studied in vision science and radiomic literature. While there are many features that can be computed within these three metrics, we have chosen to present here only a few to show the type of variations and possible high correlation with many of them.

Interpreting texture features in light of their correlation with observer performance elicits several interesting finds. First, we will consider the perceptual importance of the correlation coefficients’ signs. For positively correlating features, one possible interpretation is that larger texture values enhance observer performance. Negatively correlating features can be interpreted in the opposite way: larger values of these features hinder observer performance. Following this logic, we can now interpret the perceptual implications of our results for DBT acquisition configurations and filtering.

Our findings suggest that sparse sampling tends to reduce and increase the estimated values of concave-down and concave-up texture features, respectively. The view aliasing artifacts due to sparse sampling appears sharp and streak like resulting in heightened variations in the image. This results in an increase in values of concave-up features such as entropy which captures the randomness and decrease in concave-down features such as GLN.

In addition, our texture results for Wiener filtered images also corroborate the suggested perceptual importance of image texture. As our LROC studies indicate, application of an adaptive Wiener filter improves observer performance for our specific task. Similarly, positively correlating (concave-down) texture features also experience an increase in magnitude as a result of applying the Wiener filter. Based on our previous logic, this should imply an increase in observer performance, which is precisely what we found. Likewise, negatively correlating (concave-up) texture features decrease after applying the filter, which also implies an increase in observer performance as well.

The human observer detection experiments were conducted with medical physicists or engineers and not radiologists. The underlying argument for this is that varying strategies (acquisition/filtering) impacts the visibility of targets as seen through the quantum and anatomic (clutter) noise. This is what is being primarily tested (following a training from which the observer understands the shape and nature of the target). Our assumption is that ’once a signal is visible’ a trained radiologist will find them in a real setting.

Of the thirteen texture features that we present here, a significant number (9) of features (highlighted in the Table 4) show a very high correlation ($|r|>0.85$). These include homogeneity, entropy, contrast, complexity, busyness, GLN, RLN, run percentage and short run emphasis. Two others show very good correlation with a correlation coefficient close to 0.85. These include energy and long run emphasis. The correlations remain high even in the presence of and with significant changes in quantum noise. Two of the presented features show low correlation. These are GLCM correlation and NGTDM coarseness. One of the texture parameter, GLCM correlation is one of the least correlating parameter with AUC. The correlation length of streak-like artifact is longer than with quantum noise. The parameter GLCM correlation does not appear to be responding to changes in image artifacts seen in lower projection numbers. This results in an overall poorer correlation with human observer performance with changing projection number.

We examined possible variations in our results by changing free parameters in our texture feature calculations. This include window side of the lattice ROI ($20 \times 20$ to $100 \times 100$), pixel separation (increasing d value) and number of quantization levels (256 to 32). Considering space limitations, these results are not presented in the paper. However, these investigations showed that even though the magnitude of texture values change with these variations, the trends observed remains same and follows the human observer performance changes. Thus these changes would not change the main conclusions and results presented here. investigations into how the physics of image formation interacts with texture estimates when changing these variables would form part of our future work.

## Conclusions

With simulated but realistic digital tomographic breast images, we have shown that image texture features are sensitive to a number of system design and processing parameters. Here processing could be pre or post reconstruction filters as well as different image reconstruction methods. Thus the variations in these texture features often called ’radiomic features’ in medical imaging, is not a function of object inhomogeneity and structure (or genetic signatures manifested in the image). These features are more sensitive to image noise resulting from system geometry, reconstruction and image processing algorithms (all are vendor dependent). Thus using image texture as radiomic features must be done with extreme caution.

A more important result in this paper is the identification that variations in several well known texture features estimated in digital images correlate with human observer detection-localization performance for signals embedded in them. Extensive literature in vision science has associated several texture features with human’s ability to discriminate between images. However, to date there has been no work that points to their impact on ability to detect and localize signals in digital images. This understanding is of key significance in several areas including medical imaging and radiology where humans (or sometimes machines) search for signals/targets whose average properties may be known. It can also be of significance in images generated for defense and security applications. We illustrated that several easily computable image texture features can give insight into how the noise properties generated by system design/algorithms may impact detection-localization benefits in these images. Image processing and system changes (like detector blurring or changes in sampling) changes the magnitude of correlated noise within the image which influences the human observer’s ability to detect signals embedded in them. We examined if several readily computable texture features respond to these changes.

Our results confirm that several texture features that fall under the three well known texture metrics GLCM, NGTDM and RLM show high correlation with task-based signal detection performance in DBT images by human observers. Our preliminary investigations show that several other texture features (not reported here) from all three metrics (GLCM, NGTDM and RLM) also show high correlation to human observer studies. Our goal was to present a few popular features widely seen in vision science literature. We have highlighted some features that show high and robust correlation. These correlations stay significant against change in image noise due to geometry or processing algorithms—showing the potential utility of these methods to be used as surrogates for finding trends in task based performance assessments in medical imaging. These texture features can give reliable trends in task-based image quality that often requires tools like mathematical model observers which are computationally intensive or difficult to apply directly to clinical images. This points to the high significance of the results presented in this paper. Our findings have the potential to open new avenues in the development of practical task based assessment tools where reliable, qualitative trends would be deemed sufficient to compare imaging geometries and algorithms.

Since our work relies on simulated realistic images with signal locations known to the experimenter (but unknown to the observer), we were able to correlate the localization and detection benefits simultaneously when the observer makes decision on a given test image for signal presence. Our results show concrete evidence of the correlation of some second order texture features to human visual perception. These high correlations remain robust for varying system configurations and noise levels. While the current study tested detection of low contrast masses, these same texture features might not show high correlation for other signal types like small microcalcifications (high frequency signals). These questions are part of our ongoing investigations. The insights offered in this work could allow fast algorithmic approaches to examining detection benefits of a newly designed clinical system or algorithm. It can also offer feedback in system and algorithm designs with a goal to improve perceptual benefits^27^. Besides understanding perception in medical images, our results could benefit multiple areas such as artificial intelligence, machine learning and computer vision. In all these domains, significant benefits including transparency and simplicity can be achieved by considering the physics of image formation and basic image science concepts^53^.
